# Rapamycin as an Adjunctive Therapy for NLRC4 Associated Macrophage Activation Syndrome

**DOI:** 10.3389/fimmu.2018.02162

**Published:** 2018-09-24

**Authors:** Julie Barsalou, Annaliesse Blincoe, Isabel Fernandez, Dorothée Dal-Soglio, Lorie Marchitto, Silvia Selleri, Elie Haddad, Aissa Benyoucef, Fabien Touzot

**Affiliations:** ^1^Department of Immunology-Rheumatology, Department of Pediatric, CHU Sainte Justine, Montréal, QC, Canada; ^2^Immunology Laboratory, CHU Sainte Justine, Montréal, QC, Canada; ^3^Microbiology, Infectiology and Immunology Department, Université de Montréal, Montréal, QC, Canada; ^4^CHU Sainte Justine Research Center, Montréal, QC, Canada; ^5^Pathology and Cellular Biology Department, CHU Sainte Justine, Université de Montréal, Montréal, QC, Canada

**Keywords:** macrophage activation syndrome, NLRC4, inflammasome, mTOR, rapamycin, IL-18, IL-1β

## Abstract

Gain of function (GOF) mutations affecting the inflammasome component NLRC4 are known to cause early-onset macrophage activation syndrome (MAS) and neonatal enterocolitis. Here we report a patient with a NLRC4 GOF mutation presenting with neonatal MAS efficiently treated with a combination of anakinra and rapamycin. Through *in vitro* studies, we show that rapamycin reduces both IL-1β and IL-18 secretion by the patient's phagocytic cells. The reduction of cytokine secretion is associated with a reduction of caspase-1 activation regardless of the pathogen- or danger-associated molecular patterns triggering the activation of the inflammasome. This study suggests that patients with inherited auto-inflammatory disorders could benefit from an adjunctive therapy with rapamycin.

## Introduction

**Figure 1:**
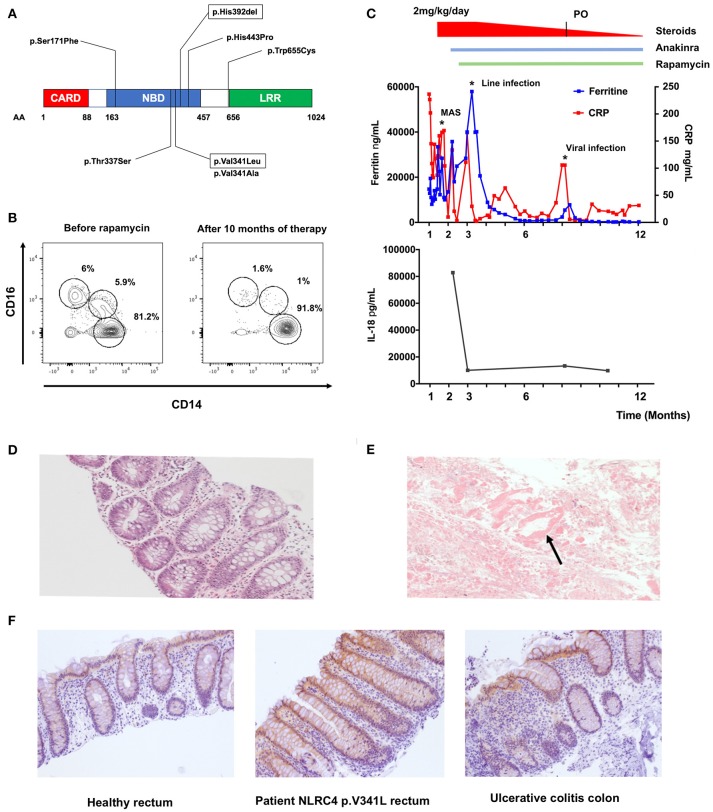
Effect of rapamycin treatment *in vivo* on a patient with pVal341Leu NLRC4 mutation. **(A)** Reported NLRC4 mutations associated with macrophage activation syndrome are located in the Nucleotide Binding Domain. Mutation p.Val341Leu and p.His392del are framed. **(B)** Evaluation of circulating monocytes subsets in the patient peripheral blood before and after initiation of therapy by flow cytometry. **(C)** Ferritin, C-reactive protein (CRP) and IL-18 levels in sera of the patient. MAS (macrophage activation syndrome). Line infection was caused by staphylococcus epidermidis. Viral infection was rhinopharyngitis without microbial documentation. **(D)** Hematoxylin and eosin staining of rectal biopsy from the patient. **(E)** Stool microscopy. Arrow point to necrotic intestinal mucosa. **(F)** IL-18 staining of rectal biopsy from healthy control (left panel), patient with NLRC4 p.V341L mutation (middle panel) and colon biopsy of a patient with ulcerative colitis (left panel).

Our patient is a male infant, born at 35 + 4 weeks of gestation to non-consanguineous parents. He was admitted to the intensive care unit at 12 days of age with profuse bloody diarrhea, weight loss, severe metabolic acidosis and acute renal failure. He developed a vasoplegic shock necessitating intubation, ventilation and inotropic support. The patient developed features of macrophage activation syndrome (MAS)/Hemophagocytic Lymphohistiocytosis (HLH) with: (i) prolonged fever >38.5°C, (ii) hepatosplenomegaly, (iii) anemia (hemoglobin 9.7g/dL) and thrombocytopenia (platelets 34 × 10^9^/L), (iv) hypertriglyceridemia (triglycerides 4.8mmol/L), (v) hyperferritinemia (14,7000 ng/mL), and (vi) haemophagocytosis on bone marrow biopsy. He also presented with three remarkable features (i) a macular erythematous rash that slowly resolved and was replaced by a reticulo-livedoid rash, (ii) a marked hypereosinophilia (up to 2.4 × 10^9^/L) and (iii) no significant elevation of HLA-DR/CD8^+^ T-cells on lymphocyte immunophenotyping. Stool microscopy showed presence of necrotic intestinal mucosa with absence of inflammatory cells (Figure 1E). Rectal biopsy demonstrated the presence of eosinophils with no significant inflammatory cell infiltrate nor architectural changes (Figure 1D). Immunohistochemistry staining performed with anti-IL18 antibody (HPA003980, Sigma) revealed moderate IL-18 staining of surface epithelium and crypts (Figure 1F). Immune work-up demonstrated global T cell lymphopenia with a balanced subpopulation. Expression of perforin and CD107a expression on NK cells was normal. Circulating FOXP3^+^CD25^+^CD127^low^CD4^+^ T-cells were within normal range. A dihydrorhodamine reduction assay was normal. Subsequent genetic analysis identified the presence of a *de novo* heterozygous mutation in the nucleotide binding domain (NBD) of NLRC4 (c.1021G>C, p.Val341Leu). This mutation is an alternate substitution of a previously reported mutation (p.Val341Ala) in a boy who presented with NLRC4 associated MAS (1).

**Figure 2:**
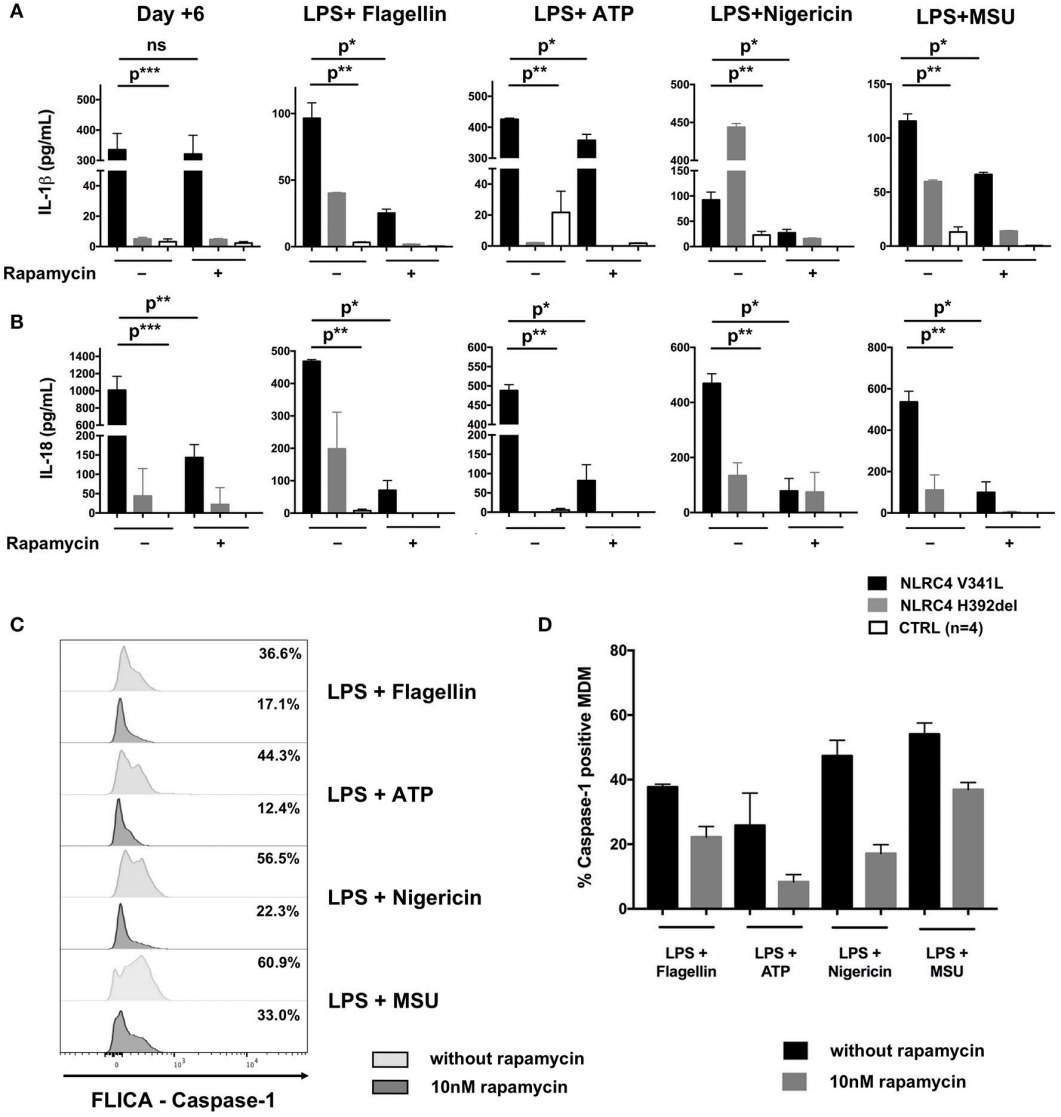
Effect of rapamycin *in vitro* in macrophage-derived monocytes from a patient with NLRC4 GOF mutations. CD14+ monocytes from patients with p.Val341Leu mutation (3 samples in duplicate) and p.His392del mutation (2 samples in duplicates) in NLRC4, and healthy controls (*n* = 4, 4 samples in duplicates) were differentiated into macrophages with or without rapamycin. Supernatant were collected at day+6 after differentiation and after stimulation of differentiated macrophage with NLRP3 (ATP, nigericin, MSU) or NLRC4 (lipotransfected flagellin) activators. IL-1β **(A)** and IL-18 **(B)** were analyzed by ELISA. **(C)** Active caspase-1 in MDM from the NLRC4 patient with p.Val341Leu mutation treated or not with 10 nM rapamycin quantified by caspase-1 FLICA after stimulation with NLRP3 (ATP, nigericin, MSU) or NLRC4 (lipotransfected flagellin) activators. **(D)** Active caspase-1 in MDM from patients with p.Val341Leu mutation (2 samples) and p.His392del mutation in NLRC4 (2 samples in duplicates),treated or not with 10 nM rapamycin quantified by caspase-1 FLICA after stimulation with NLRP3 (ATP, nigericin, MSU) or NLRC4 (lipotransfected flagellin) activators. p* < 0.05; p** < 0.01, p*** < 0.001; ns not significant (Man-Whitney *T-*test or Wilcoxon paired *T*-test).

We demonstrated the spontaneous activation of NLRC4 *in vitro* as evidenced by the high level of IL-1β (317 ± 168 pg/mL vs. 5 ± 2 pg/mL, *p* = 0.0007) and IL-18 (205 ± 175 pg/mL vs. 0 pg/mL, *p* < 0.0001) secretion by unstimulated monocyte-derived macrophages (MDM) as compared to healthy controls (HC) (Figure 2A). Of note, we also observed higher secretion of IL-1β and IL-18 by the patient's MDM after sequential stimulation by lipopolysaccharide (LPS) and different NLRP3 [adenosine triphosphate (ATP), monosodium urate crystal (MSU) or nigericin] and NLRC4 (lipotransfected flagellin) activators as compared to MDM from HC (Figure 2A).

The patient was initially treated with IV methylprednisone (2 mg/kg/day) and anakinra (up to 15 mg/kg/day). We decided to introduce rapamycin as a steroid-sparing agent reasoning that this drug could (i) potentiate the action of anakinra through autophagy induction and (ii) counteract the effect of IL-18 on T-cells through mTOR inhibition (2–5). The targeted trough concentration of rapamycin was 10–15 ng/mL. The patient showed clinical response to the therapy within a week, allowing weaning of steroids, establishment of enteral feeds and weight gain. The C-reactive protein and ferritin dropped to 15–30 mg/L and 250–300 ng/mL respectively (Figure 1C). Relative proportions of CD14^+^CD16^−^ “classical,” CD14^+^CD16^+^ “intermediate” and CD14^low^CD16^+^ “non-classical” monocytes also normalized under therapy (Figure 1B) (6). Intriguingly, we documented a marked decrease of IL-18 (from 82844 to 9776 ng/mL, Figure 1C) in the patient's plasma, whereas IL-1β levels remained undetectable throughout the follow-up. This feature suggested a specific effect of rapamycin on IL-18 secretion. Previous reports of patients with NLRC4 GOF have indeed shown chronic elevation of serum IL-18 despite aggressive anti-inflammatory and IL-1 receptor blockade therapy (7). Of note, a rectal biopsy performed 6 months after initiation of rapamycin therapy showed persistent IL-18 staining (data not shown).

## Background

Inflammasomes are innate immune complexes that respond to pathogen- and danger-associated molecular patterns (PAMP and DAMP) through caspase-1 activation and interleukin (IL)-1β and IL-18 secretion (8). Recently, gain of function (GOF) mutations affecting the NLRC4 inflammasome component have been reported to cause early-onset recurrent fever and MAS (1, 9).

Most reported mutations are located in the NBD of the protein (Figure 1A). The mutations are thought to disrupt the interaction between the leucine-rich repeat (LRR) domain and the NBD, facilitating NLRC4 conformational changes and its steady activation (10).

IL-1β blockade is effective in most inherited disorders associated with inflammasome dysregulation. However, the precise role of IL-1β in the development of MAS remains controversial (11). Interestingly, NLRC4 GOF has emphasized the important role of IL-18 in the development of MAS (12). In this disease, IL-18 secretion depends not only on phagocytic cells but also on epithelial cells (12, 13). Management of MAS associated with NLRC4 GOF mutations remains challenging. Minimal or absent response to anakinra has been described in patients with early onset disease (7, 11, 14) Recombinant IL-18BP (tadekinig-α) has shown promising results in a patient with NLRC4 GOF mutation and is under evaluation in a phase 3 study (NCT03113760) (7). Allogeneic hematopoietic stem cell transplantation (HSCT) is the treatment of choice for primary HLH, however to date there have been no published report published of such treatment in patients with NLRC4 GOF mutations (15). HSCT may indeed cure the hyperinflammation driven by the immune system, but it is unlikely to have significant impact on the intestinal disease nor the IL-18 hypersecretion by the intestinal epithelial cells (12, 13).

## Discussion

The results presented here constitute the first *in vivo* and *in vitro* evidence of the anti-inflammatory effect of mTOR inhibitors in inherited inflammasome disorders. Patients with NLRC4 GOF mutations remain difficult to manage due to the severity of disease and the partial response to therapy (1, 9). We therefore decided to introduce rapamycin as adjunctive therapy reasoning that this drug could (i) potentiate the action of anakinra through autophagy induction and (ii) counteract the effect of IL-18 on T-cells through mTOR inhibition (2–4). The clinical and biochemical improvement of our patient mirrored the effects of rapamycin seen on MDM from the patient *in vitro*. While rapamycin had no remarkable effect on the spontaneous secretion of IL-1β by the patient's MDM during the first 6 days of differentiation culture prior to further stimulation, it significantly reduced their IL-18 secretion (Figure 2A). This could be explained by (i) different mechanisms through which rapamycin counteracts IL-1β and IL-18 cleavage and secretion or (ii) differences in epigenetic and transcriptional regulation of the *IL1B* and *IL18* loci. Of note, rapamycin also significantly reduced the secretion of both IL-1β and IL-18 by the patient's MDM after sequential activation with LPS plus activators of the NLRP3 and NLRC4 inflammasomes (Figures 2A,B). This reduction in cytokine secretion was associated with reduction of caspase-1 activation—as evidenced by FLICA-Caspase 1 assay—regardless of the PAMP or DAMP involved in inflammasome activation (Figures 2B,C). Interestingly, the same effect of rapamycin was observed in MDM derived from the patient with the p.His392del mutation and in MDM from healthy donors (Figure 2), showing that the effect of rapamycin was not restricted to the reported patient. Unfortunately, we could not demonstrate a reduction of IL-18 secretion by rapamycin in rectal epithelial cells because of the non-quantitative nature of the immunohistochemistry staining.

Altogether, these clinical and *in vivo* and *in vitro* biological data demonstrate for the first time that rapamycin is an interesting adjunctive therapy for patients with NLRC4 GOF mutations though reduction of both IL-1β and IL-18 secretion by phagocytic cells. This reduction of cytokine secretion is associated with a reduction of caspase-1 activation but the precise mechanism through which rapamycin inhibits inflammasome activation remains to be determined (3, 13). This effect could involve diverse counter-regulatory pathways in phagocytes and perhaps also in intestinal epithelial cells (3, 13).

## Concluding remarks

We report herein a patient with a NLRC4 GOF mutation presenting with neonatal MAS efficiently treated with a combination of anakinra and rapamycin. The mTOR inhibitor reduced secretion of both IL-1β and IL-18 by the patient's phagocytic cells, regardless of the PAMP or DAMP triggering the inflammation, through reduction of caspase-1 activation. Our report paves the way for the use of mTOR inhibitors in the management of patients presenting with inherited inflammasome disorders.

### Methods

#### Macrophage differentiation

Monocytes were isolated from PBMCs by negative selection using the EasySep™ Human Monocyte Isolation Kit (19058, Stem Cell Technology) according to the manufacturer's instructions. Monocytes were differentiated in Stemspan Medium with 100 ng/mL of recombinant human Granulocyte-Macrophage Colony-Stimulating Factor (Peprotech) for 6 days with or without 10 nM rapamycin (R8781, Sigma) (added at day 0 of culture and at day 3 without changing the medium).

#### Macrophage secretion assay

After 6 days of differentiation, MDM were stimulated for 3 h by lipopolysaccharide (LPS) followed by stimulation with different activators of NLRP3 or NLRC4 inflammasome (2.5 mM ATP for 45 min; 250 μg/ml MSU, 2.5 μM nigericin, and 1 μg/ml of lipotransfected flagellin, all for 4 h) in presence or absence of 10 nM rapamycin. The caspase-1 activity was assessed by flow cytometry using caspase-1 FLICA (ICT098, Bio-Rad) according to the manufacturer's instructions.

IL-1β and IL-18 were measured in plasma from patient and culture medium by ELISA (88-7261-88 and BMS267-2 from eBioscience). IL-18 analysis in plasma of patients required a 25-fold dilution to allow measurement in the linear part of the standard curve.

### Immunohistochemistry

IL-18 immunohistochemistry staining was performed with anti-IL18 antibody (HPA003980, Sigma) according to standard procedures.

### Monocytes subset analysis

Monocytes subset analysis was performed on total fresh blood using the following antibodies: anti CD16 PeCy7, anti-CD14 VioBlue, anti-HLA-DR FITC from BD biosciences.

### Statistical analysis

Statistical analysis was performed with Graphpad Prism software (version 6.0; GraphPad, La Jolla, CA). Man-Whitney *T*-test was used for comparison of unpaired values, whereas Wilcoxon paired *T*-test was used for comparison of paired values.

## Ethics statement

Consent: This study was carried out in accordance with the recommendations of CHU Sainte-Justine Institutional Review Board with written informed consent from all subjects. All subjects gave written informed consent in accordance with the Declaration of Helsinki. The protocol was approved by the CHU Sainte-Justine Institutional Review Board. Written informed consent was obtained from the parents for the publication of this case reports.

## Author contributions

JB and ABl took care of the patient, collected data and participated in the manuscript redaction. IF, DD-S, SS, and LM performed experiments and critically read the manuscript. EH took care of the patient, collected data, and critically read the manuscript. ABe designed the study, performed experiments, and critically read the manuscript. FT designed the study, performed experiments, wrote the manuscript, and took care of the patient.

### Conflict of interest statement

The authors declare that the research was conducted in the absence of any commercial or financial relationships that could be construed as a potential conflict of interest.
